# Quality of life in a randomized trial of early closure of temporary ileostomy after rectal resection for cancer (EASY trial)

**DOI:** 10.1002/bjs.10680

**Published:** 2017-11-23

**Authors:** J. Park, A. K. Danielsen, E. Angenete, D. Bock, A. C. Marinez, E. Haglind, J. E. Jansen, S. Skullman, A. Wedin, J. Rosenberg

**Affiliations:** ^1^ Department of Surgery, Institute of Clinical Sciences, Sahlgrenska Academy, University of Gothenburg, Scandinavian Surgical Outcomes Research Group Sahlgrenska University Hospital Östra Gothenburg Sweden; ^2^ Department of Surgery Skaraborgs Sjukhus Skövde Sweden; ^3^ Department of Gastroenterology, Herlev and Gentofte Hospital University of Copenhagen Herlev Ringvej Herlev Denmark; ^4^ Department of Surgery Nordsjaellands Hospital Hillerød Denmark

## Abstract

**Background:**

A temporary ileostomy may reduce symptoms from anastomotic leakage after rectal cancer resection. Earlier results of the EASY trial showed that early closure of the temporary ileostomy was associated with significantly fewer postoperative complications. The aim of the present study was to compare health‐related quality of life (HRQOL) following early versus late closure of a temporary ileostomy.

**Methods:**

Early closure of a temporary ileostomy (at 8–13 days) was compared with late closure (at more than 12 weeks) in a multicentre RCT (EASY) that included patients who underwent rectal resection for cancer. Inclusion of participants was made after index surgery. Exclusion criteria were signs of anastomotic leakage, diabetes mellitus, steroid treatment, and signs of postoperative complications at clinical evaluation 1–4 days after rectal resection. HRQOL was evaluated at 3, 6 and 12 months after resection using the European Organisation for Research and Treatment of Cancer (EORTC) questionnaires QLQ‐C30 and QLQ‐CR29 and Short Form 36 (SF‐36®).

**Results:**

There were 112 patients available for analysis. Response rates of the questionnaires were 82–95 per cent, except for EORTC QLQ‐C30 at 12 months, to which only 54–55 per cent of the patients responded owing to an error in questionnaire distribution. There were no clinically significant differences in any questionnaire scores between the groups at 3, 6 or 12 months.

**Conclusion:**

Although the randomized study found that early closure of the temporary ileostomy was associated with significantly fewer complications, this clinical advantage had no effect on the patients' HRQOL. Registration number: NCT01287637 (https://www.clinicaltrials.gov).

## Abstract 2

No different after early ileostomy closure

## Introduction

Low anterior resection with total mesorectal excision is regarded as one of the optimal surgical treatments for potentially curable carcinoma of the rectum^1^, ^2^. Because of the low anastomosis close to the pelvic floor, patients often receive a temporary ileostomy at the time of the resection to reduce the risk of symptomatic anastomotic dehiscence^3^ and its clinical consequences^3^, ^4^, ^5^, ^6^. However, studies^4^, ^7^, ^8^, ^9^ have reported considerable morbidity related to the temporary ileostomy, with complication rates of up to 50 per cent. Most patients with a temporary ileostomy have the stoma for at least 3 months, and it is not unusual for it to be left in place much longer. For some patients the stoma becomes permanent^10^.

Data regarding quality of life (QOL) in patients receiving a diverting stoma as part of their rectal cancer treatment are limited^11^, ^12^, ^13^, ^14^. In prospective studies it has been suggested that patients with a stoma may suffer from impaired health‐related quality of life (HRQOL)^15^, which may improve at stoma closure^2^. Complications such as stoma leakage, parastomal skin irritation, dietary restrictions, retraction and prolapse of the stoma have been reported to have significant impact on the patient's daily life^12^.

The aim of the present study was to compare HRQOL at 3, 6 and 12 months after rectal resection for cancer in a multicentre RCT comparing early *versus* late closure of a temporary ileostomy (EASY trial)^16^, ^17^.

## Methods

The EASY trial was designed as a randomized multicentre trial^16^ comparing early with late closure of a temporary ileostomy regarding risk of complications. Screening for and inclusion of participants was made after index surgery (total mesorectal excision for rectal cancer including creation of a temporary ileostomy). Exclusion criteria were diabetes mellitus, ongoing steroid treatment, signs of postoperative complications at clinical evaluation 1–4 days after rectal resection and inability to understand the Danish or Swedish language. Patients with no adverse signs were invited to participate and, after informed consent, underwent further investigation with contrast‐enhanced CT or a flexible endoscopy of the rectum, or both, performed 6–8 days after stoma creation to ensure that no patient with signs of anastomotic leakage was included. Patients were randomized to either the intervention group with early closure (day 8–13 after stoma creation) or the control group with late closure (more than 12 weeks after stoma creation) of the ileostomy.

The primary endpoint of the study was the mean number of complications after rectal resection and up to 12 months; these results have been published previously^17^. The present paper reports the secondary endpoints, HRQOL and QOL, at 3, 6 and 12 months after the index operation.

The study was approved in Denmark by the Science Ethics Committee for the Capital Region (H‐1‐2010‐113) and in Sweden by the Regional Ethics Approval Committee in Göteborg (Dnr 064‐2011). Before inclusion, patients were informed about the study and all participating patients returned a signed consent form.

The project was approved by the Data Protection Agency in Denmark, and by the Personal Data Representative at Sahlgrenska University Hospital, Göteborg, Sweden.

The protocol was registered at https://www.clinicaltrials.gov (NCT01287637) before patient inclusion.

### Patients

Eight hospitals in Denmark and Sweden participated in the study, but three centres (with a total of 8 patients) were excluded as they failed to maintain a screening log. Consenting patients were asked to complete questionnaires at 3, 6 and 12 months after stoma creation (rectal resection). The questionnaires included the 36‐item Short Form 36 (SF‐36®; Rand Corporation, Santa Monica, California, USA) and the European Organisation for Research and Treatment of Cancer Quality of Life Questionnaire (EORTC QLQ) CR29 and C30. Data regarding demographic details, tumour stage and height, chemoradiotherapy and all complications within 12 months of surgery were registered in case report forms.

### Randomization

Consenting patients who fulfilled the inclusion criteria were randomized either to the intervention group with early closure of the ileostomy or to the control group with late closure. Randomization was executed in computer‐generated blocks of six. The randomization was performed on the surgical ward using sequentially numbered thick, opaque and sealed envelopes. Blinding of the intervention was not possible.

### Health‐related quality‐of‐life instruments

#### *Short Form 36*

SF‐36® is a generic tool that evaluates patients' self‐reported quality of life^18^, ^19^. It consists of 36 items that measure eight dimensions of health on a multi‐item scale, including social and physical function. The scoring scale ranges from 0 to 100, with lower scores indicating worse health. The instrument has been validated, and for comparison in this study a Swedish reference population was used^20^.

#### *EORTC QLQ‐C30 and QLQ‐CR29*

The EORTC QLQ‐C30 is a questionnaire developed to assess QOL in patients with cancer, and consists of five functional scales (physical, role, cognitive, emotional and social), three symptom scales (fatigue, pain and nausea, and vomiting), one global health status and QOL scale, and six single‐item measures (dyspnoea, insomnia, appetite loss, constipation, diarrhoea and financial difficulties)^21^. A high score on the functional scale represents a high level of functioning, whereas a high score on the symptom scale represents a high level of symptoms.

The EORTC QLQ‐CR29 was designed for use in patients undergoing treatment for colorectal cancer. It was derived from the EORTC QLQ‐C38 questionnaire, as there was a need for an update in the colorectal module^22^. The questions assess disease symptoms, side‐effects of treatment, body image, future perspective, and sexual function and interest. Both questionnaires have been validated internationally and were available in Danish and Swedish versions^22^, ^23^.

All questionnaires were administered at 3, 6 and 12 months after stoma creation.

### Statistical analysis

The study was part of a RCT with power calculated for the primary endpoint. Group sizes in the EASY trial were set to 72 patients per group to evaluate complication rates^16^, ^17^. In Krouse *et al*.^24^ a minimally important difference of 8 units was used for the different scales of SF‐36®. Physical and mental component scores had a standard deviation (s.d.) of up to 15 units, and the eight specific scales had s.d. values in the range 17–33. With group sizes of 72 or 55 and a s.d. of 17 or 15 respectively, there will be 80 per cent power to detect a true difference of 8 units.

The questionnaires SF‐36®^25^, EORTC QLQ‐C30^26^ and QLQ‐CR29^23^ were scored according to the methods recommended by the developers; missing data were handled as instructed in the scoring manuals. Before analysis, based on the literature and previous data, the authors chose to present the functional scales and global health status/QOL of the EORTC QLQ‐C30 questionnaire, and the functional scales (urinary frequency, stool frequency and body image) of the EORTC QLQ‐CR29 questionnaire. At 12‐month follow‐up, several patients (20 in the intervention group and 16 in the control group) were, by mistake, given an incomplete questionnaire in which questions 16–30 of the EORTC QLQ‐C30 were missing. Before analysis, the decision was made to include physical and role functioning, as these functional scales are scored using questions 1–15 in accordance with the EORTC manual. No other scales or items were analysed for these patients in the EORTC QLQ‐C30 at 12‐month follow‐up. However, EORTC QLQ‐CR29 and SF‐36® were analysed at 12‐month follow‐up. As the distribution of the incomplete questionnaires was independent of the observable characteristics of the patients who received them, interpreting the missing data as being completely random is reasonable and imputation was considered unnecessary.

Owing to the characteristics of the data, the different scales of SF‐36® and EORTC were summarized by median (i.q.r.) values, and group comparisons were made using the Wilcoxon rank sum test and the two‐sample Hodges–Lehmann estimator.

SF‐36® scores were compared with those in a general Swedish reference population^20^. For each of the eight scales, the individual levels were compared with age‐matched (17–34, 35–49, 50–64, and 65 or more years) mean levels from the reference population. The proportion of patients with values below the reference levels was compared between the intervention and control group using a χ^2^ test. The software packages SPSS® version 23 (IBM, Armonk, New York, USA), SAS® version 9.4 (SAS Institute, Cary, North Carolina, USA) and R version 3.2.3^27^ were used for statistical analysis. Scores for SF‐36®, EORTC QLQ‐C30 and QLQ‐CR29 were derived and summarized by median (i.q.r.) values.

## Results

**Figure 1:**
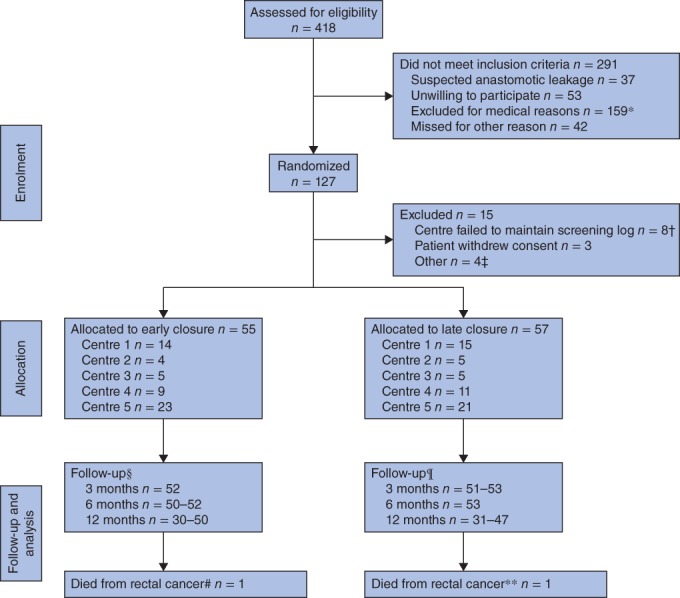
Participant flow diagram, as in the EASY trial^17^. *Paralytic ileus (24), Hartmann procedure with intersphincteric dissection (16), delayed postoperative recovery (15), perioperative complications (7), other infection (5), reoperation (7), high stoma output (5), pulmonary embolism (1), ulcerative colitis (1), extensive cancer disease (3), cardiovascular disease (2), language difficulty (5), diabetes (28), permanent or no stoma (29), steroid treatment (3), other (8). †Centre 6 (2), centre 7 (3), centre 8 (3). ‡Allocated to early closure, but not possible to perform surgery within 8–13 days (1); early closure outside the study (2); randomized, but no further information available (1). §3 months: Short Form 36 (SF‐36®) (52), European Organisation for Research and Treatment of Cancer (EORTC) QLQ‐C30 (52), EORTC QLQ‐CR29 (52); 6 months: 3 months: SF‐36® (52), EORTC QLQ‐C30 (50), EORTC QLQ‐CR29 (50); 12 months: 3 months: SF‐36® (50), EORTC QLQ‐C30 (30), EORTC QLQ‐CR29 (50). ¶3 months: SF‐36® (53), EORTC QLQ‐C30 (51), EORTC QLQ‐CR29 (52); 6 months: 3 months: SF‐36® (53), EORTC QLQ‐C30 (53), EORTC QLQ‐CR29 (53); 12 months: SF‐36® (47), EORTC QLQ‐C30 (31), EORTC QLQ‐CR29 (47). #Missing from follow‐up at 12 months. **Patient did not have closure; missing from follow‐up at 12 months

The EASY trial assessed 418 patients for eligibility. After exclusion of 291 patients, 127 patients were randomized (*Fig*. 1). A further 15 patients were excluded, eight from three centres that were excluded from the study as they failed to maintain a screening log. In summary, 112 patients were included from February 2011, with the last follow‐up in November 2015. Some 55 patients in the intervention (early closure) group and 57 in the control (late closure) group were available for analysis. There were no violations of the randomization. Except for a larger proportion of women in the intervention group, baseline demographic characteristics and clinical data were similar in the two groups (*Table* 1).

**Table 1:** Baseline and preoperative characteristics of patients randomized to early or late closure

	Early closure (*n* = 55)	Late closure (*n* = 57)
Age (years)*	67 (36–82)	67 (39–81)
Sex ratio (F : M)	31 : 24	21 : 36
BMI (kg/m^2^)*	24 (17–32)	23 (19–35)
Co‐morbidity	23 (42)	24 (42)†
Ischaemic heart disease	5	8
Hypertension	17	13
COPD	2	2
Renal disease	0	0
Other	9‡	4§
Radiotherapy	16 (29)	16 (28)
Long‐term	5 (9)	5 (9)
Adjuvant chemotherapy	22 (40)	23 (40)
Marital status		
Single	5 (9)	9 (16)
Married	42 (76)	43 (75)
Widowed	8 (15)	5 (9)
Higher education	34 (62)	37 (65)
Employed		
Yes	25 (45)	25 (44)
No	29 (53)¶	32 (56)
Smoker	6 (11)	4 (7)
No. of pack years*	30 (16–30)#	26 (20–50)¶
Alcohol intake > 60 g/day	0 (0)	0 (0)¶
Lower border of tumour (cm from anal verge)		
5–9	27 (49)	24 (42)
10–15	27 (49)	33 (58)
> 15	1 (2)	0 (0)
UICC clinical stage**		
I	12 (22)	19 (33)
II	21 (38)	13 (23)
III	18 (33)	20 (35)
IV	3 (5)	1 (2)
Method of evaluation of anastomosis before ileostomy closure		
CT	14 (25)	19 (33)
Rectoscopy	14 (25)	10 (18)
CT + rectoscopy	27 (49)	28 (49)
Total length of hospital stay†† (days) *	14 (11–42)	14 (7–44)

Values in parentheses are percentages unless indicated otherwise;

* values are median (range).

† Data missing for two patients.

‡ Asthma (2), depression (1), idiopathic thrombocytopenic purpura (1), lymphoma (1), Waldenström's macroglobulinaemia (1), osteoporosis (1), Sjögren syndrome (1), thyrotoxicosis (1).

§ Depression (1), hyperlipidaemia (1), hypothyroidism (1), meningioblastoma (1).

¶ Data missing for one patient.

# Data missing for three patients.

** Data missing for one patient in each group; in addition, three patients in the late closure group had T0 N0 M0 disease and were therefore not classified.

†† For both index surgery and loop ileostomy closure. COPD, chronic obstructive pulmonary disease; UICC, International Union Against Cancer. Table reproduced with permission from Wolters Kluwer Health Inc. Danielsen AK, Park J, Jansen JE, Bock D, Skullman S, Wedin A *et al*. Early closure of a temporary ileostomy in patients with rectal cancer: a multicenter randomized controlled trial. Ann Surg 2017; **265 (2)**: 284–290. https://journals.lww.com/annalsofsurgery/.

Questionnaire response rates were generally around 90 (range 82–95) per cent, excluding the EORTC QLQ‐C30 at 12‐month follow‐up, owing to missing questions as described in the Methods section (*Fig*. 1).

SF‐36® scores were similar between the two groups, with no differences in the physical component score or the mental component score, but significant differences in role physical, bodily pain and mental health at 3, 12 and 12 months respectively (*Table* 2). All dimensions in SF‐36® improved over time. At 3 months, a majority of patients in both groups scored values below mean levels in the reference population^20^, especially regarding role physical. At 12 months, 52–85 per cent of the patients scored higher than the reference group, with physical functioning scoring the highest among the dimensions.

**Table 2:** SF‐36® scores at 3, 6 and 12 months after rectal resection

	3 months			6 months			12 months		
	Median (i.q.r.)	H–L*	*P* †	Median (i.q.r.)	H–L*	*P* †	Median (i.q.r.)	H–L*	*P* †
Physical functioning									
Early	90 (75–95)	0 (−5, 5)	0·646	90 (81·7–100)	0 (−5, 5)	0·630	95 (70–100)	0 (0, 5)	0·322
Late	90 (80–95)			90 (80–95)			95 (90–100)		
Role physical									
Early	75 (50–96·9)	12·5 (0, 18·8)	0·025	81·3 (50–100)	6·3 (0, 18·8)	0·140	81·3 (56·3–100)	0 (−6·3, 6·3)	0·718
Late	62·5 (43·8–75)			75 (50–93·8)			87·5 (75–100)		
Bodily pain									
Early	80 (52–100)	0 (−10, 0)	0·858	74 (62–100)	0 (−16, 0)	0·264	79 (51–100)	0 (0, 20)	0·035
Late	74 (62–100)			84 (63–100)			100 (74–100)		
General health									
Early	71·6 (52–88·5)	−5 (−15, 2)	0·139	77 (56–87)	0 (−10, 5)	0·820	74·5 (45–92)	5 (−5, 16·8)	0·279
Late	77 (67–87)			77 (65–87)			82 (72–87)		
Vitality									
Early	62·5 (43·8–81·3)	4·2 (−6·3, 12·5)	0·441	68·8 (50–81·3)	0 (−12·5, 6·3)	0·796	68·8 (50–81·3)	6·3 (0, 12·5)	0·196
Late	68·8 (56·3–81·3)			68·8 (56·3–81·3)			75 (62·5–87·5)		
Social functioning									
Early	75 (62·5–100)	0 (−12·5, 0)	0·468	87·5 (62·5–100)	0 (0, 0)	0·976	87·5 (62·5–100)	0 (0, 12·5)	0·415
Late	87·5 (75–100)			87·5 (62·5–100)			100 (75–100)		
Role emotional									
Early	83·3 (58·3–100)	0 (0, 8·3)	0·345	87·5 (66·7–100)	0 (0, 0)	0·923	95·8 (66·7–100)	0 (0, 0)	0·697
Late	83·3 (50–100)			83·3 (75–100)			95·8 (75–100)		
Mental health									
Early	80 (55–90)	5 (−5, 10)	0·217	80 (60–90)	−5 (−10, 5)	0·291	80 (60–90)	10 (0, 15)	0·020
Late	85 (65–90)			85 (70–95)			85 (75–95)		
Mental component score									
Early	52·5 (40·7–58·6)	1 (−2·6, 5)	0·588	54·4 (42·8–58·6)	0·2 (−3, 3·9)	0·939	54·1 (42·6–58·5)	2·5 (−0·7, 6·3)	0·105
Late	53 (44·8–57·8)			54·6 (46·9–57·5)			56·6 (52·9–59·2)		
Physical component score									
Early	51·8 (40·9–58·2)	−0·5(−3·8, 3·4)	0·823	53·3 (43·3–57·1)	−0·2 (−3·6, 3)	0·900	54·1 (44·5–59)	1·6 (−1, 6·1)	0·281
Late	51·2 (46·9–54·8)			52·2 (45·8–57·9)			56·8 (51–59·4)		

* Two‐sample Hodges–Lehmann (H–L) estimator with 95 per cent asymptotic confidence limits in parentheses. SF‐36®, Short Form 36.

† Wilcoxon rank sum test for difference between early and late closure.

EORTC QLQ‐C30 and QLQ‐CR29 scores were comparable between intervention and control groups. Emotional functioning was lower in the early closure group at 3 and 6 months, but similar to the late closure group at 12 months (*Table* 3). No statistically significant differences were seen in the dimensions of the QLQ‐CR29 questionnaire (*Table* 4).

**Table 3:** EORTC QLQ‐C30 scores at 3, 6 and 12 months after rectal resection

	3 months			6 months			12 months		
	Median (i.q.r.)	H–L*	*P* †	Median (i.q.r.)	H–L*	*P* †	Median (i.q.r.)	H–L*	*P* †
Global quality of life									
Early	75 (50–83·3)	0 (−8·3, 8·3)	0·941	66·7 (50–83·3)	0 (−8·3, 8·3)	0·961	83·3 (50–91·7)	0 (−16·7, 8·3)	0·889
Late	66·7 (58·3–83·3)			66·7 (66·7–83·3)			83·3 (66·7–91·7)		
Physical functioning									
Early	93·3 (73·3–100)	0 (0, 6·7)	0·634	93·3 (80–100)	0 (−6·7, 0)	0·433	93·3 (73·3–100)	0 (−13·3, 0)	0·137
Late	93·3 (73·3–100)			93·3 (80–100)			100 (80–100)		
Role functioning									
Early	83·3 (66·7–100)	−16·7 (−16·7, 0)	0·066	100 (66·7–100)	0 (0, 0)	0·503	100 (66·7–100)	0 (0, 0)	0·793
Late	66·7 (50–100)			83·3 (66·7–100)			100 (66·7–100)		
Emotional functioning									
Early	83·3 (66·7–100)	8·3 (0, 16·7)	0·023	83·3 (66·7–100)	−8·3 (−16·7, 0)	0·031	91·7 (66·7–100)	0 (−8·3, 0)	0·409
Late	91·7 (83·3–100)			91·7 (75–100)			91·7 (83·3–100)		
Cognitive functioning									
Early	100 (83·3–100)	0 (0, 0)	0·447	83·3 (83·3–100)	0 (−16·7, 0)	0·131	100 (66·7–100)	0 (0, 0)	0·652
Late	100 (83·3–100)			100 (83·3–100)			100 (83·3–100)		
Social functioning									
Early	83·3 (66·7–100)	0 (0, 0)	0·583	83·3 (66·7–100)	0 (0, 0)	0·882	83·3 (66·7–100)	0 (−16·7, 0)	0·142
Late	83·3 (66·7–100)			83·3 (66·7–100)			100 (66·7–100)		

* Two‐sample Hodges–Lehmann (H–L) estimator with 95 per cent asymptotic confidence limits in parentheses. EORTC, European Organisation for Research and Treatment of Cancer.

† Wilcoxon rank sum test for difference between early and late.

**Table 4:** EORTC QLQ‐CR29 scores for selected functions at 3, 6 and 12 months after rectal resection

	3 months			6 months			12 months		
	Median (i.q.r.)	H–L*	*P* †	Median (i.q.r.)	H–L*	*P* †	Median (i.q.r.)	H–L*	*P* †
Urinary frequency									
Early	16·7 (0–33·3)	0 (0, 16·7)	0·323	16·7 (0–50)	0 (−16·7, 0)	0·353	16·7 (0–33·3)	0 (0, 16·7)	0·268
Late	16·7 (0–50)			16·7 (8·3–41·7)			33·3 (0–50)		
Stool frequency									
Early	33·3 (16·7–50)	33·3 (16·7, 33·3)	< 0·001	33·3 (16·7–50)	16·7 (0, 33·3)	0·068	33·3 (16·7–50)	0 (0, 16·7)	0·611
Late	0 (0–16·7)			16·7 (0–66·7)			33·3 (16·7–50)		
Body image									
Early	88·9 (66·7–100)	0 (−11·1, 0)	0·715	88·9 (77·8–100)	0 (0, 11·1)	0·364	94·4 (77·8–100)	0 (0, 0)	0·502
Late	77·8 (66·7–100)			88·9 (66·7–100)			100 (88·9–100)		

* Two‐sample Hodges–Lehmann (H–L) estimator with 95 per cent asymptotic confidence limits in parentheses. EORTC, European Organisation for Research and Treatment of Cancer.

† Wilcoxon rank sum test for difference between early and late closure.

## Discussion

In this RCT no significant differences were observed in HRQOL within 12 months after rectal resection for cancer when early and late closure of temporary ileostomy were compared. Global QOL generally improved later in the follow‐up period (6–12 months), and at 12 months the results were comparable, not only between the two groups but also with respect to both age‐matched reference populations^28^, ^29^ and previous findings^30^. Although there was a tendency for improvement over time in global QOL and role functioning, no clinically significant changes were seen in QLQ‐C30 scores. The definition of a clinically significant change over time was based on the suggestion^31^ that a difference of 5–10 points be considered a ‘ little change’ and a difference of 10–20 points a ‘ moderate change’. When comparing the SF‐36® scores with Swedish reference data^20^, a general improvement was seen during the follow‐up interval.

No baseline data for preoperative assessment were available, owing to the fact that the patients were enrolled and randomized after the rectal resection. This is considered a weakness in the evaluation of HRQOL. Even though reference data were available, there was no opportunity to investigate the development and changes in HRQOL from preoperative to postoperative values. In addition, considering the length of time needed for recovery after rectal cancer surgery, follow‐up at 18 and 24 months might have been of value to observe any further development and potential improvement in HRQOL.

The lack of difference between the two groups differs somewhat from the findings of previous prospective studies^2^, ^13^, which have reported that patients with a temporary stoma may suffer from impaired HRQOL in comparison with patients who had undergone a similar operation but without a covering stoma, such as a high anterior resection. However, the results of previous studies are contradictory. A Danish study^32^ suggested that a temporary stoma left patients with feelings of uncertainty, that closure of the stoma was seen as a crucial event, and that knowing the date for closure was important. A prospective study^11^ found improved global QOL following stoma reversal after low anterior resection for rectal cancer, and physical function, as measured by both the QLQ‐C30 and SF‐36® questionnaires, was also significantly improved. However, a prospective interview study^14^ reported no change in QOL following closure of a temporary stoma after rectal cancer surgery, and the patient's personality rather than clinical variables has a strong and lasting influence on QOL^33^. As suggested in a review article^34^ from 2011, closure of the stoma and prompt redirection of intestinal contents to the rectum 3–6 months after rectal resection might be considered to be a cause of negative impact on patients' physical, social and psychological health for several months. Gastrointestinal symptoms, such as increased stool frequency, urgency, diarrhoea and persistent problems including low anterior resection syndrome, do not occur in the presence of a temporary stoma. Consequently, stoma reversal might result in the appearance of these symptoms. Although this was not observed in the present study, this could be because of the small size of the cohort. In the COLOR II trial^35^, patients with rectal cancer appeared to need a longer time to recover compared with those with colonic cancer. This could perhaps explain the lack of difference between the groups in the present study, as both involved extensive surgery for rectal cancer, where timing of the stoma closure is not reflected in the HRQOL follow‐up.

The EASY trial^17^ found that it was safe and advantageous to close the loop ileostomy early in patients with no clinical or radiological signs of anastomotic leakage. However, the present study did not find a link between this clinical advantage and patients' HRQOL.

## References

[1] Heald RJ . Total mesorectal excision is optimal surgery for rectal cancer: a Scandinavian consensus. Br J Surg 1995; 82: 1297–1299. 7489148 10.1002/bjs.1800821002

[2] O'Leary DP, Fide CJ , Foy C , Lucarotti ME . Quality of life after low anterior resection with total mesorectal excision and temporary loop ileostomy for rectal carcinoma. Br J Surg 2001; 88: 1216–1220. 11531870 10.1046/j.0007-1323.2001.01862.x

[3] Matthiessen P , Hallböök O , Rutegård J , Simert G , Sjödahl R . Defunctioning stoma reduces symptomatic anastomotic leakage after low anterior resection of the rectum for cancer: a randomized multicenter trial. Ann Surg 2007; 246: 207–214. 17667498 10.1097/SLA.0b013e3180603024 PMC1933561

[4] Ihnat P , Gunkova P , Peteja M , Vavra P , Pelikan A , Zonca P . Diverting ileostomy in laparoscopic rectal cancer surgery: high price of protection. Surg Endosc 2016; 30: 4809–4816. 26902615 10.1007/s00464-016-4811-3

[5] Montedori A , Cirocchi R , Farinella E , Sciannameo F , Abraha I. Covering ileo‐ or colostomy in anterior resection for rectal carcinoma. Cochrane Database Syst Rev 2010; (5)CD006878. 20464746 10.1002/14651858.CD006878.pub2 PMC12721704

[6] Hüser N , Michalski CW , Erkan M , Schuster T , Rosenberg R , Kleeff J *et al* Systematic review and meta‐analysis of the role of defunctioning stoma in low rectal cancer surgery. Ann Surg 2008; 248: 52–60. 18580207 10.1097/SLA.0b013e318176bf65

[7] Gessler B , Haglind E , Angenete E . Loop ileostomies in colorectal cancer patients – morbidity and risk factors for nonreversal. J Surg Res 2012; 178: 708–714. 22940030 10.1016/j.jss.2012.08.018

[8] Hallböök O , Matthiessen P , Leinsköld T , Nyström PO , Sjödahl R . Safety of the temporary loop ileostomy. Colorectal Dis 2002; 4: 361–364. 12780582 10.1046/j.1463-1318.2002.00398.x

[9] Gessler B , Haglind E , Angenete E . A temporary loop ileostomy affects renal function. Int J Colorectal Dis 2014; 29: 1131–1135. 25026894 10.1007/s00384-014-1949-0

[10] den Dulk M , Smit M , Peeters KC , Kranenbarg EM , Rutten HJ , Wiggers T *et al* A multivariate analysis of limiting factors for stoma reversal in patients with rectal cancer entered into the total mesorectal excision (TME) trial: a retrospective study. Lancet Oncol 2007; 8: 297–303. 17395102 10.1016/S1470-2045(07)70047-5

[11] Camilleri‐Brennan J , Steele RJ . Prospective analysis of quality of life after reversal of a defunctioning loop ileostomy. Colorectal Dis 2002; 4: 167–171. 12780610 10.1046/j.1463-1318.2002.00352.x

[12] Gooszen AW , Geelkerken RH , Hermans J , Lagaay MB , Gooszen HG . Quality of life with a temporary stoma: ileostomy *vs*. colostomy. *Dis* *Colon Rectum* 2000; 43: 650–655. 10.1007/BF02235581 10826426

[13] Tsunoda A , Tsunoda Y , Narita K , Watanabe M , Nakao K , Kusano M . Quality of life after low anterior resection and temporary loop ileostomy. *Dis* *Colon Rectum* 2008; 51: 218–222. 10.1007/s10350-007-9101-7 18172730

[14] Siassi M , Hohenberger W , Lösel F , Weiss M . Quality of life and patient's expectations after closure of a temporary stoma. Int J Colorectal Dis 2008; 23: 1207–1212. 18685854 10.1007/s00384-008-0549-2

[15] Anaraki F , Vafaie M , Behboo R , Maghsoodi N , Esmaeilpour S , Safaee A . Quality of life outcomes in patients living with stoma. Indian J Palliat Care 2012; 18: 176–180. 23439841 10.4103/0973-1075.105687 PMC3573471

[16] Danielsen AK , Correa‐Marinez A , Angenete E , Skullman S , Haglind E , Rosenberg J ; SSORG (Scandinavian Outcomes Research Group) . Early closure of temporary ileostomy – the EASY trial: protocol for a randomised controlled trial. BMJ Open 2011; 1: e000162. 10.1136/bmjopen-2011-000162 PMC3191573 22021780

[17] Danielsen AK , Park J , Jansen JE , Bock D , Skullman S , Wedin A *et al* Early closure of a temporary ileostomy in patients with rectal cancer: a multicenter randomized controlled trial. Ann Surg 2017; 265: 284–290. 27322187 10.1097/SLA.0000000000001829

[18] Anderson RT , Aaronson NK , Wilkin D . Critical review of the international assessments of health‐related quality of life. Qual Life Res 1993; 2: 369–395. 8161975 10.1007/BF00422215

[19] Ware JE Jr, Sherbourne CD . The MOS 36‐item short‐form health survey (SF‐36). I. Conceptual framework and item selection. Med Care 1992; 30: 473–483. 1593914

[20] Taft C , Karlsson J , Sullivan M . Performance of the Swedish SF‐36 version 2.0. Qual Life Res 2004; 13: 251–256. 15058805 10.1023/B:QURE.0000015290.76254.a5

[21] Aaronson NK , Ahmedzai S , Bergman B , Bullinger M , Cull A , Duez NJ *et al* The European Organization for Research and Treatment of Cancer QLQ‐C30: a quality‐of‐life instrument for use in international clinical trials in oncology. J Natl Cancer Inst 1993; 85: 365–376. 8433390 10.1093/jnci/85.5.365

[22] Gujral S , Conroy T , Fleissner C , Sezer O , King PM , Avery KN *et al*; European Organisation for Research and Treatment of Cancer Quality of Life Group. Assessing quality of life in patients with colorectal cancer: an update of the EORTC quality of life questionnaire. Eur J Cancer 2007; 43: 1564–1573. 17521904 10.1016/j.ejca.2007.04.005

[23] Whistance RN , Conroy T , Chie W , Costantini A , Sezer O , Koller M *et al*; European Organisation for Research and Treatment of Cancer Quality of Life Group. Clinical and psychometric validation of the EORTC QLQ‐CR29 questionnaire module to assess health‐related quality of life in patients with colorectal cancer. Eur J Cancer 2009; 45: 3017–3026. 19765978 10.1016/j.ejca.2009.08.014

[24] Krouse RS , Herrinton LJ , Grant M , Wendel CS , Green SB , Mohler MJ *et al* Health‐related quality of life among long‐term rectal cancer survivors with an ostomy: manifestations by sex. J Clin Oncol 2009; 27: 4664–4670. 19720920 10.1200/JCO.2008.20.9502 PMC2754912

[25] Ware JE , Kosinski M , Dewey JE . How to Score Version 2 of the SF‐36® Health Survey. QualityMetric: Lincoln, 2000.

[26] Fayers PM , *Aaronson NK, Bjordal K, Groenvold M, Curran D, Bottomley A; EORTC Quality of Life Group*. The EORTC QLQ‐C30 Scoring Manual (3rd edn). European Organisation for Research and Treatment of Cancer: Brussels, 2001.

[27] R Core Team . R: A Language and Environment for Statistical Computing. R Foundation for Statistical Computing: Vienna, 2016.

[28] Michelson H , Bolund C , Nilsson B , Brandberg Y . Health‐related quality of life measured by the EORTC QLQ‐C30 – reference values from a large sample of Swedish population. Acta Oncol 2000; 39: 477–484. 11041109 10.1080/028418600750013384

[29] Schwarz R , Hinz A . Reference data for the quality of life questionnaire EORTC QLQ‐C30 in the general German population. Eur J Cancer 2001; 37: 1345–1351. 11435063 10.1016/s0959-8049(00)00447-0

[30] Neuman HB , Patil S , Fuzesi S , Wong WD , Weiser MR , Guillem JG *et al* Impact of a temporary stoma on the quality of life of rectal cancer patients undergoing treatment. Ann Surg Oncol 2011; 18: 1397–1403. 21128000 10.1245/s10434-010-1446-9

[31] Osoba D , Rodrigues G , Myles J , Zee B , Pater J . Interpreting the significance of changes in health‐related quality‐of‐life scores. J Clin Oncol 1998; 16: 139–144. 9440735 10.1200/JCO.1998.16.1.139

[32] Danielsen AK , Soerensen EE , Burcharth K , Rosenberg J . Impact of a temporary stoma on patients' everyday lives: feelings of uncertainty while waiting for closure of the stoma. J Clin Nurs 2013; 22: 1343–1352. 23279240 10.1111/jocn.12011

[33] Siassi M , Weiss M , Hohenberger W , Losel F , Matzel K . Personality rather than clinical variables determines quality of life after major colorectal surgery. Dis Colon Rectum 2009; 52: 662–668. 19404072 10.1007/DCR.0b013e31819ecf2e

[34] Taylor C , Morgan L . Quality of life following reversal of temporary stoma after rectal cancer treatment. Eur J Oncol Nurs 2011; 15: 59–66. 20667779 10.1016/j.ejon.2010.06.002

[35] Andersson J , Angenete E , Gellerstedt M , Angeras U , Jess P , Rosenberg J *et al* Health‐related quality of life after laparoscopic and open surgery for rectal cancer in a randomized trial. Br J Surg 2013; 100: 941–949. 23640671 10.1002/bjs.9144 PMC3672685

